# Magnetically Modified Bentonite for Optimized Erythromycin Removal via RSM and DFT Analysis

**DOI:** 10.3390/molecules30081792

**Published:** 2025-04-16

**Authors:** Ying-Chieh Hung, Yu-Qi Wu, Ru-Hau Ye, Hsiu-Min Hung, Gui-Bing Hong

**Affiliations:** Department of Chemical Engineering and Biotechnology, National Taipei University of Technology, No. 1, Sec. 3, Zhongxiao E. Rd., Taipei City 106, Taiwan; hungyc@ntut.edu.tw (Y.-C.H.);

**Keywords:** adsorption, bentonite, erythromycin, magnetization, response surface methodology, density functional theory

## Abstract

Erythromycin (ERY), an antibiotic widely used in human and veterinary medicine, persists in the environment due to its low degradability, accumulating in wastewater and soil. This study presents a novel adsorbent synthesized by magnetically modifying calcined natural bentonite with Fe_3_O_4_ nanoparticles to enhance ERY removal. The modification increased the surface area, with the highest adsorption observed at pH 11. Adsorption studies revealed that the Dubinin–Radushkevich isotherm model and pseudo-first-order kinetic model best described the adsorption behavior. Response surface methodology (RSM) was employed to optimize key parameters, including adsorbent dosage, temperature, and contact time. The quadratic model indicated optimal conditions of 41.9 mg adsorbent, 29.1 °C, and 9.6 h of contact time, yielding a maximum ERY removal efficiency of 96.2%. Density functional theory (DFT) analysis provided a molecular-level understanding of the adsorption mechanism, identifying strong interactions between ERY, Fe_3_O_4_, and bentonite. The theoretical binding energy aligns with experimental results, confirming the role of magnetic modification in promoting ERY adsorption. This study demonstrates a promising approach for mitigating ERY contamination in aqueous environments.

## 1. Introduction

Emerging contaminants (ECs) are residual chemical substances found in the environment due to household, agricultural, and industrial activities. These contaminants include pesticides, fertilizers, heavy metals, pharmaceutical active compounds (PhACs), and personal care products (PPCPs), among others [1]. Despite their typically low concentrations, ECs are of significant concern due to their persistence in the environment and potential to cause harm. They are not easily decomposed, which allows them to accumulate in soil, groundwater, and oceans. Over time, these contaminants can enter the human body through the food chain, posing serious health risks, including reproductive and endocrine disorders in humans and wildlife [2].

Erythromycin (ERY), a macrolide antibiotic derived from the fermentation of Streptomyces erythraea, is widely used in both human and veterinary medicine due to its broad-spectrum efficacy and relatively low side effects. However, its complex structure makes chemical synthesis difficult, leading to its predominant production via fermentation [3]. ERY and its derivatives are among the most frequently used antibiotics, and as a result, they are often detected in municipal and industrial wastewater, particularly from pharmaceutical manufacturing processes [4]. The persistence of ERY in the environment is problematic; it is resistant to degradation, leading to its accumulation in wastewater, soil, and other ecosystems. This accumulation can have detrimental effects on both environmental and human health [5].

A variety of technologies have been developed to remove antibiotic pollutants like ERY from the environment, including adsorption [6], advanced oxidation processes [7], photocatalytic degradation [8], electrocoagulation [9], membrane treatment [10], and biodegradable methods [11]. Among these, adsorption stands out as a particularly effective and straightforward method due to its potential for regeneration and the availability of various adsorbent materials. Effective adsorbents, such as biochar, activated carbon, zeolite, and clay, are characterized by high adsorption capacity, ease of regeneration, and environmental friendliness.

Clay materials, including kaolin, bentonite, and fly ash, are abundant, cost-effective, and readily available, making them attractive options for adsorbent development [12]. However, the quality of natural clay materials can be inconsistent, necessitating further processing to enhance their properties and suitability as commercial adsorbents. Bentonite, a clay mineral derived from volcanic ash, is particularly noteworthy for its high cation exchange capacity and water absorption properties, which make it an effective adsorbent [13]. Recent reviews have highlighted the potential of bentonite in environmental remediation [14,15]. Various modification methods have been explored to enhance the adsorption performance of bentonite-based composite materials, including acid/alkali washing [16,17], thermal treatment [18,19], and hybridization [20,21]. Among these, thermal treatment is widely recognized as an effective technique for modifying bentonite, particularly in solid–liquid adsorption applications such as fixed-bed adsorption systems.

Despite the extensive use of bentonite in environmental remediation, its application in ERY removal remains underexplored, particularly in combination with advanced modification techniques. The novelty of this study lies in the following:

(1) The development of a magnetically modified bentonite (MCB) for enhanced ERY adsorption: While bentonite has been studied as an adsorbent, its modification via calcination followed by magnetization is an innovative approach. This process not only enhances adsorption performance but also enables easy separation and recyclability, minimizing secondary environmental pollution;
(2) The application of response surface methodology (RSM) for adsorption optimization: Unlike many adsorption studies that examine parameter effects individually, this work employs RSM to systematically optimize adsorption conditions, providing deeper insights into interactive effects and process efficiency;
(3) Investigation of adsorption mechanisms using Density Functional Theory (DFT): Most studies on antibiotic adsorption focus on experimental data without in-depth molecular-level interaction analysis. This study employs DFT calculations to elucidate the binding mechanisms between ERY and MCB, providing a theoretical foundation for understanding adsorption behavior.

By integrating experimental and computational approaches, this study offers a comprehensive analysis of ERY adsorption onto MCB, demonstrating its potential as an effective and sustainable adsorbent for antibiotic removal from wastewater.

## 2. Discussion

### 2.1. Adsorbent Characterization

**Figure 1:**
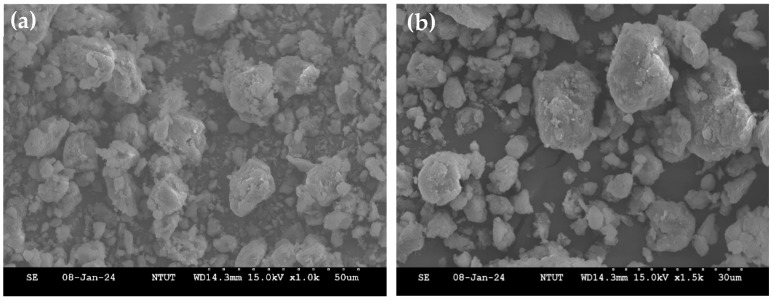
SEM images of (**a**) natural bentonite and (**b**) calcined bentonite (CB).

**Figure 2:**
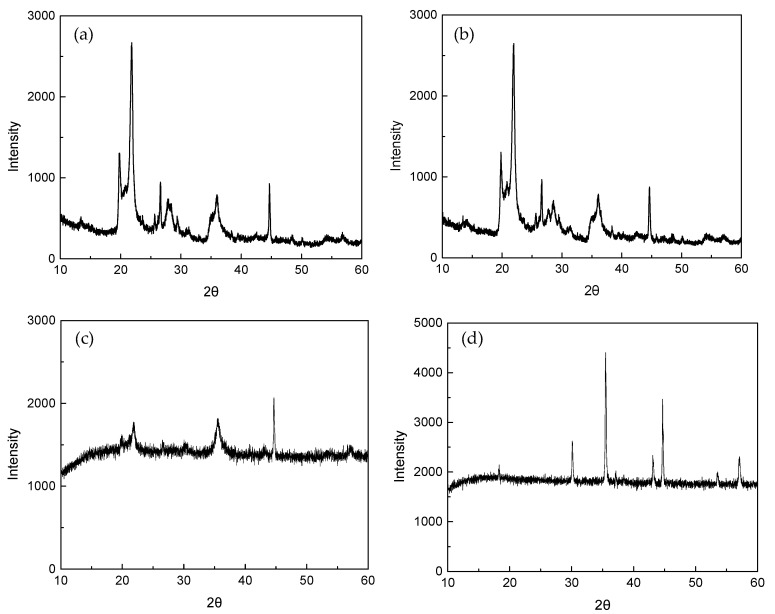
XRD patterns of (**a**) natural bentonite, (**b**) calcined bentonite (CB), (**c**) magnetic calcined bentonite (MCB), and (**d**) Fe_3_O_4_.

The calcination of natural bentonite, also known as dehydroxylation, is intended to remove hydroxyl groups from its structure, thereby activating the material and enhancing its reactivity [22]. As observed in the SEM micrographs (Figure 1), both natural bentonite and bentonite, calcined at 500 °C (CB), exhibit an amorphous and dispersed morphology. The XRD analysis results (Figure 2) indicate that the crystal structure and crystallinity of natural bentonite and CB remain largely unchanged, suggesting that calcination at high temperatures does not compromise the thermal stability of bentonite. However, a slight reduction in peak intensity is observed in CB, which may be attributed to partial dehydroxylation. After magnetization, the XRD spectrum of MCB (Figure 2c) shows additional diffraction peaks corresponding to Fe_3_O_4_ (Figure 2d), particularly the characteristic peak at 2θ ≈ 35.38°, confirming the successful incorporation of Fe_3_O_4_ particles into the bentonite matrix. Compared to CB, the XRD peaks of MCB exhibit further broadening and decreased intensity, indicating a reduction in crystallinity due to the introduction of Fe_3_O_4_ nanoparticles. These structural changes suggest that while calcination preserves the overall crystal integrity of bentonite, magnetization alters its crystalline nature by forming a composite material with Fe_3_O_4_ infiltration.

**Figure 3:**
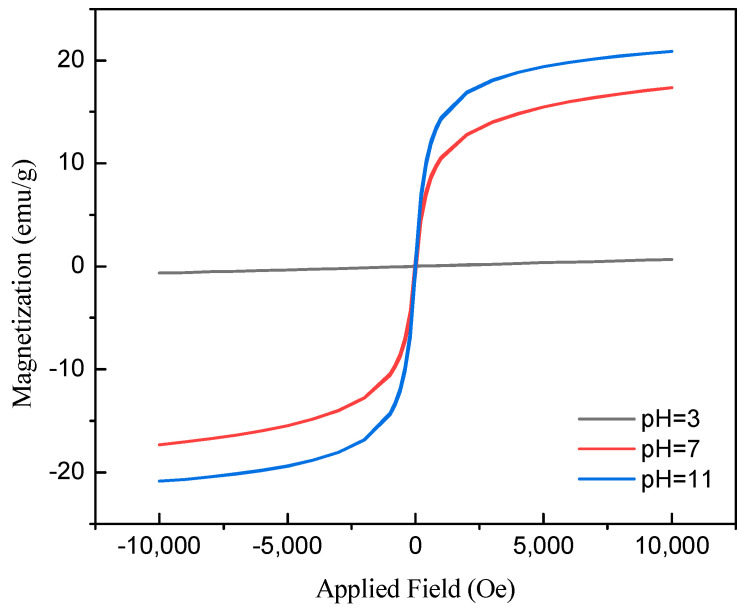
Magnetization versus applied magnetic field for MCB in different pH conditions.

Figure 3 illustrates the magnetic properties of the adsorbent (MCB) following magnetization at pH levels of 3, 7, and 11, as measured by a Superconducting Quantum Interference Device Vibrating Sample Magnetometer (SQUID-VSM, MPMS7, Quantum Design Inc., San Diego, CA, USA). The data clearly show that the adsorbent prepared at pH 11 in an alkaline environment possesses the strongest magnetic properties, followed by the adsorbent prepared at pH 7, with the weakest magnetic response observed at pH 3.

**Figure 4:**
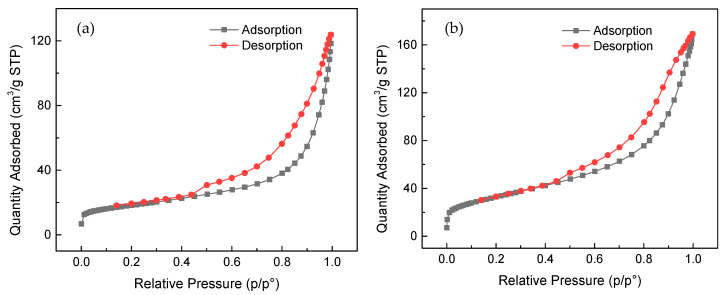
N_2_ adsorption–desorption isotherms of (**a**) CB and (**b**) MCB.

Figure 4 presents the nitrogen adsorption–desorption isotherms of CB and MCB. According to the IUPAC classification, both CB and MCB display type IV isotherms with H3 hysteresis loops, indicative of the presence of larger pores and slit-like voids. Table 1 summarizes the specific surface area, pore volume, and pore diameter of the adsorbents. The specific surface area of CB is 41.4 m^2^/g, which significantly increases to 61.6 m^2^/g after magnetization. This moderate increase suggests that the Fe_3_O_4_ modification improves structural dispersion and prevents particle aggregation, leading to a higher accessible surface area [23]. The pore volume of CB is 0.003 cm^3^/g, which increases to 0.008 cm^3^/g for MCB, indicating the formation of additional porosity upon Fe_3_O_4_ incorporation. Additionally, the average pore diameter decreases from 11.9 nm to 7.6 nm, likely due to partial pore blockage or structural rearrangements caused by the magnetic modification. These results confirm that the observed changes in porosity are consistent with the expected effects of Fe_3_O_4_ deposition on the bentonite structure.

**Figure 5:**
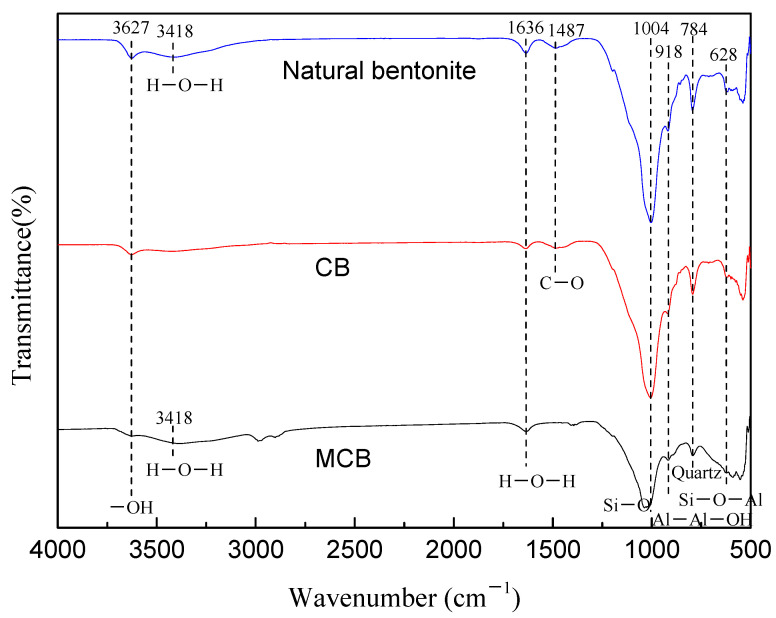
FTIR spectrum of natural bentonite, CB, and MCB.

FTIR analysis was performed in the range of 500–4000 cm^−1^ (Figure 5), and the characteristic absorption peaks were observed at approximately 3627 cm^−1^ and 3418 cm^−1^. These peaks correspond to the stretching vibrations of structural hydroxyl (-OH) groups, specifically from the silanol (Si-OH) groups in the octahedral layer of montmorillonite and the Al-OH group, respectively [24]. The peak at around 1636 cm^−1^ is attributed to the structural components of the mineral, particularly the asymmetric bending mode of H-O-H from interlayer water [25]. The region near 1004 cm^−1^ corresponds to the Si-O stretching vibrations in the tetrahedral structure [26]. A weaker absorption peak at 1487 cm^−1^ is indicative of C-O stretching, suggesting the presence of carbonate impurities [27]. Peaks at 918 cm^−1^ and 784 cm^−1^ are associated with Al-Al-OH vibrations of bentonite and Si-O stretching due to quartz, respectively [28]. After modification of the natural bentonite, changes in the asymmetric Si-O stretching vibrations were observed, with shifts and deformation occurring at 1004 cm^−1^, 790 cm^−1^, and 530 cm^−1^ in the CB and MCB samples. Additionally, modifications in the structural hydroxyl group (-OH) near 3500 cm^−1^ and the asymmetric H-O-H bending at 1636 cm^−1^ indicate the removal of water molecules and changes in the hydrophobicity of the bentonite [29].

### 2.2. Influence of Parameters on ERY Adsorption

**Figure 6:**
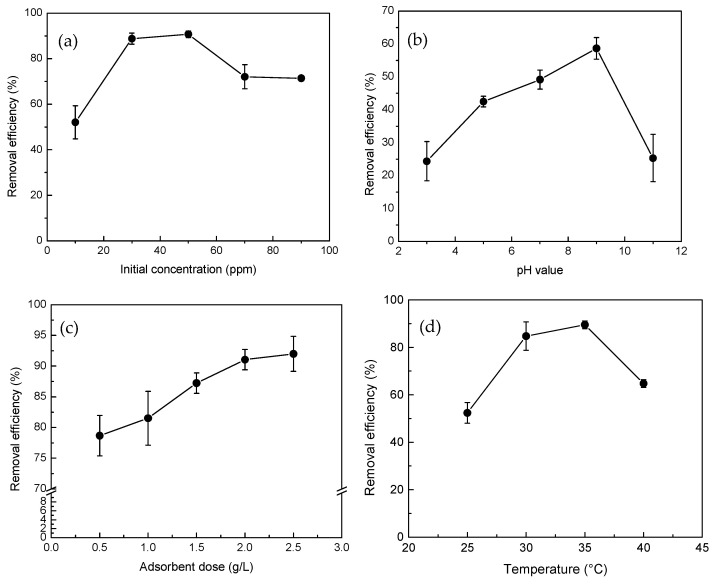
Effect of operating parameters on ERY adsorption onto MCB: (**a**) initial concentration, (**b**) pH value, (**c**) adsorbent dosage, and (**d**) adsorption temperature.

The impact of various operational parameters on the efficiency of ERY adsorption is illustrated in Figure 6. The experiments were conducted under the following conditions: an MCB dosage of 2 g/L, adsorption temperature of 30 °C, pH 9 of the original ERY solution, and a contact time of 24 h. The effect of different initial ERY concentrations (10, 30, 50, 70, and 90 ppm) on adsorption efficiency is shown in Figure 6a. The results indicate a rapid increase in ERY removal efficiency at concentrations between 10 and 30 ppm, suggesting that the ERY molecules were fully adsorbed onto the MCB surface. At concentrations between 30 and 50 ppm, the system reached adsorption equilibrium, maintaining a stable removal efficiency. However, at concentrations ranging from 50 to 90 ppm, the active adsorption sites on the MCB surface became saturated, leading to a decrease in the removal rate as excess ERY molecules could no longer be effectively adsorbed. Therefore, an initial ERY concentration of 30 ppm was selected as a fixed parameter for subsequent optimization studies using RSM.

**Figure 7:**
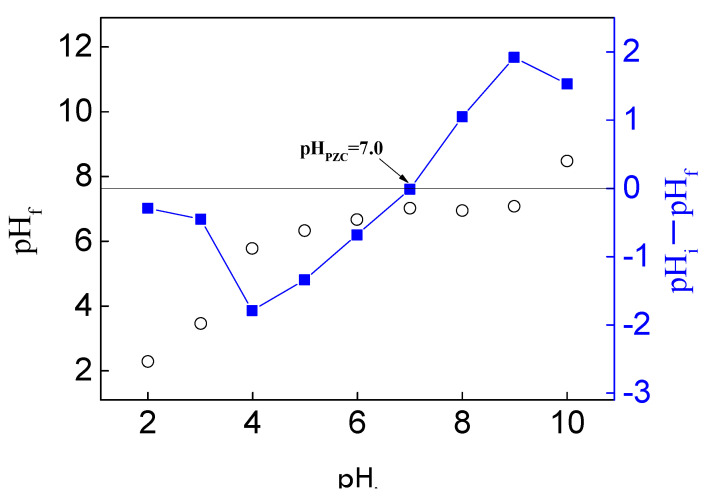
The determination of the pH_pzc_ of MCB.

The pH of the solution significantly influences the surface charge of the adsorbent and the ionization state of the adsorbate. When the adsorbent and adsorbate carry opposite charges, electrostatic attraction is enhanced, thereby increasing the adsorption capacity. Conversely, similar charges lead to reduced adsorption. This study investigated the effect of pH values ranging from 3 to 11 on ERY adsorption by MCB (initial concentration: 50 ppm, adsorbent dose: 2 g/L, temperature: 30 °C, contact time: 24 h), as shown in Figure 6b. Given that the pKa of ERY is 8.80, ERY primarily exists as a cation (ERY^+^) in neutral to acidic environments (2.0 < pH < 8.80) and as a non-ionized or zwitterionic species (ERY^±^) in alkaline conditions [30]. The pH_PZC_ (point of zero charge) of MCB was determined to be 7.0 (Figure 7), indicating that the adsorbent surface is positively charged at pH < 7.0 and negatively charged at pH > 7.0. The results demonstrate an increasing ERY removal rate within the pH range of 3 to 9, which can now be attributed to the negatively charged surface of MCB at pH > 7.0, promoting the adsorption of positively charged ERY^+^ ions through electrostatic attraction. However, a significant reduction in adsorption efficiency was observed at pH 11, as ERY exists in a non-ionized form in an alkaline environment, diminishing the electrostatic attraction and thus lowering the removal rate. Given the substantial influence of pH on ERY adsorption, pH 9 was selected as the fixed parameter for the subsequent RSM optimization.

The effect of varying adsorbent dosages (0.5, 1.0, 1.5, 2.0, and 2.5 g/L) on ERY adsorption (initial concentration: 50 ppm, original pH, temperature: 30 °C, contact time: 24 h) was also explored, with the results depicted in Figure 6c. The removal efficiency of ERY increased with higher adsorbent doses. While a higher adsorbent dose can swiftly and effectively remove contaminants, excessive dosage may lead to competition for active sites, potentially diminishing or even reducing adsorption capacity. The optimal removal efficiency of approximately 90% was achieved at an adsorbent dose of 2 g/L. Further increasing the dosage to 2.5 g/L did not result in a significant improvement in removal efficiency, likely due to adsorbent aggregation, which reduces the total surface area and subsequently decreases the adsorption effect [31].

Finally, the effect of different adsorption temperatures (25, 30, 35, and 40 °C) on ERY adsorption (initial concentration: 50 ppm, original pH, adsorbent dose: 2 g/L, adsorption time: 24 h) was investigated, as shown in Figure 6d. The results reveal that within the temperature range of 25 to 35 °C, the removal efficiency increases with temperature. However, at 40 °C, the adsorption efficiency declines, indicating that while a moderate increase in temperature can enhance ERY adsorption, excessively high temperatures may inhibit the adsorption capacity of the MCB adsorbent [30].

### 2.3. Adsorption Isotherms

The isotherm models are instrumental in elucidating the adsorption mechanism, interactions, surface properties of the adsorbent, and the affinity between the adsorbate and adsorbent by relating the concentration of antibiotics in solution to the adsorption capacity per unit mass of adsorbent. In this study, the Langmuir, Freundlich, and Dubinin–Radushkevich (D–R) isotherm models were employed to investigate the adsorption behavior of ERY onto the MCB. Adsorption experiments were conducted under optimal single-factor conditions, including an aqueous solution pH of 9, initial ERY concentrations of 10–50 ppm, a contact time of 4 h, and an adsorbent dosage of 2.0 g/L, across various temperatures (25, 30, 35, and 40 °C). The adsorption data for ERY on MCB were fitted to the Langmuir, Freundlich, and D–R isotherm models, with the corresponding results presented in Table 2. Among the models, the D–R isotherm provided the best fit for the adsorption behavior of MCB at different temperatures. The calculated sorption energy (E) from the D–R model was less than 8 kJ/mol, suggesting that the adsorption process is predominantly driven by physical adsorption [32].

### 2.4. Adsorption Kinetics

**Figure 8:**
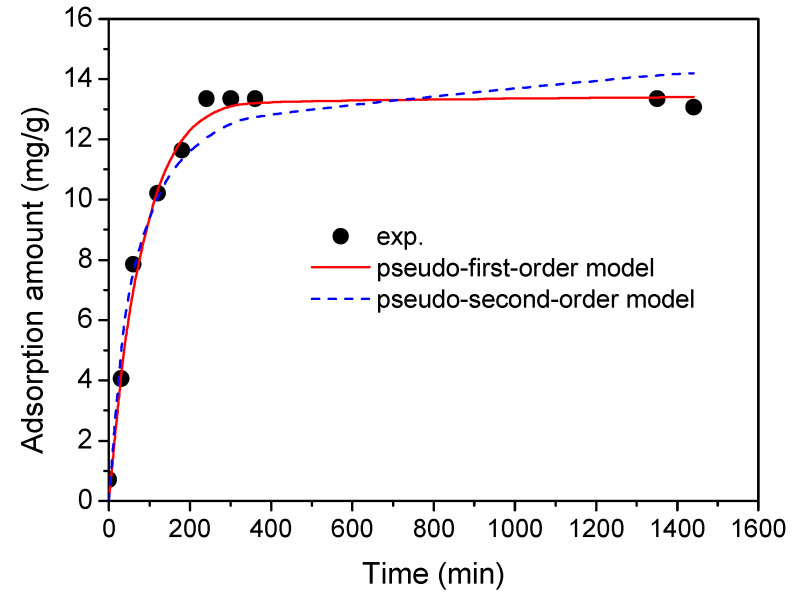
Adsorption kinetic data and correlated results.

The adsorption kinetics study was performed under optimal single-factor conditions, including a solution pH of 9, an initial ERY concentration of 30 ppm, an adsorption temperature of 35 °C, and an adsorbent dosage of 2 g/L. The results of the kinetic experiment (Figure 8) revealed that the adsorption equilibrium for ERY onto MCB was reached after 4 h, with an adsorption capacity of 13.36 mg/g. To better understand the adsorption mechanism, the kinetic data were fitted using the non-linear forms of the pseudo-first-order and pseudo-second-order kinetic models. The correlated curves and parameters are presented in Figure 8 and Table 3, respectively. The correlation coefficients (*R*^2^) were 0.989 for the pseudo-first-order model and 0.959 for the pseudo-second-order model, indicating that both models reasonably describe the experimental data. However, the *q_e_* value obtained from the pseudo-first-order model was more closely aligned with the experimental *q_exp_*, suggesting that the adsorption kinetics of ERY onto MCB are better represented by the pseudo-first-order model under the studied conditions.

### 2.5. Adsorption Optimization

The adsorption of ERY by MCB was optimized using a central composite design (CCD) within the framework of response surface methodology (RSM). The experimental conditions were fixed at a solution pH of 9 and an initial ERY concentration of 30 ppm. Three independent variables were evaluated: adsorbent dosage (10–50 mg), temperature (25–45 °C), and contact time (0–12 h). The experimental design matrix and corresponding results are presented in Table 4, demonstrating ERY removal efficiencies ranging from 2.4% to 90.5%. Among the various models tested, including linear, cubic, and quadratic models, the second-order polynomial model provided the highest *R*^2^ value, indicating a strong correlation with the experimental data and accurately capturing the relationships between the independent variables and the response. The mathematical expression of the second-order polynomial model is as follows:

Removal efficiency (%) = 73.65+ 2.47A − 16.36B + 5.48C − 11.91AB − 1.79AC − 4.76BC + 1.06A^2^ + 0.6439B^2^ − 20.82C^2^ (1)

**Figure 9:**
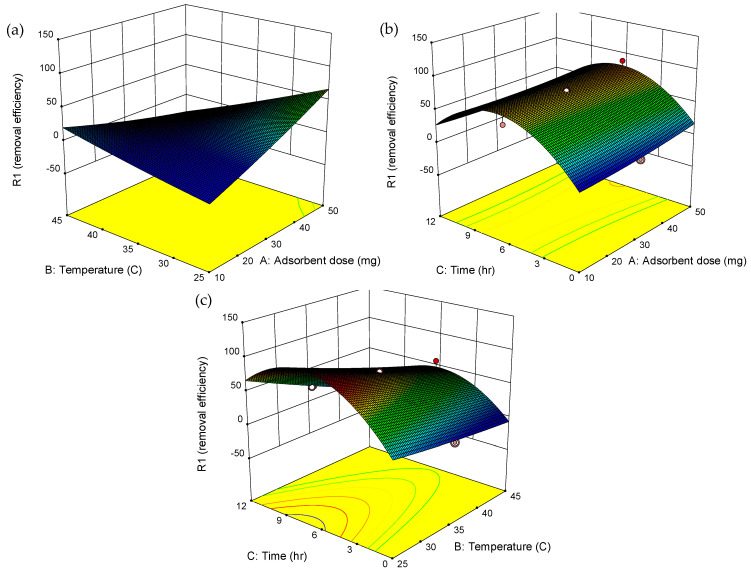
Contour and 3D surface plots showing the effects of (**a**) adsorbent dosage and temperature, (**b**) adsorbent dosage and time, and (**c**) temperature and time.

Table 5 presents the analysis of variance (ANOVA) for the second-order model, which reveals a *p*-value of 0.0004, confirming the model’s statistical significance for predicting ERY removal efficiency. The significant model terms, identified by *p*-values less than 0.05, include the linear effect of temperature (B), the interaction between adsorbent dosage and temperature (AB), and the quadratic term for contact time (C^2^), highlighting their critical roles in the adsorption process. Contour and 3D surface plots (Figure 9) illustrate the interactions between the independent variables and their influence on ERY removal. In Figure 9a, the combined effect of adsorbent dosage (A) and temperature (B) shows that increasing both factors enhances the removal efficiency. The non-linearity and distortions observed in the 3D plots (Figure 9b,c) are consistent with the ANOVA results, reflecting the complex interactions between variables. Using the optimization feature of Design-Expert software, the optimal conditions for maximum ERY removal were determined to be an adsorbent dosage of 41.9 mg, a temperature of 29.1 °C, and a contact time of 9.6 hrs. Under these optimal conditions, the predicted ERY removal efficiency was 93.7%, which closely aligned with the experimentally observed value of 96.2%, thereby validating the efficacy of the CCD approach in optimizing the adsorption process in this study.

### 2.6. DFT Calculations

#### 2.6.1. The Structure and Binding Behavior of ERY Adsorption on CB and MCB

The quantum effects of Fe_3_O_4_ nanoparticles of HOMO/LUMO positions and HOMO-LUMO gap are shown in Table 6, columns 2, 3, and 4. It can be observed that the HOMO energy and HOMO-LUMO gap demonstrated obvious size-dependent behavior as expected. The shrinking behavior of the HOMO-LUMO gap becomes convergent when the particle size exceeds that of Fe_9_O_12_ and the HOMO-LUMO gap of Fe_3_O_4_, Fe_6_O_8_, Fe_9_O_12_, and Fe_12_O_16_ are 2.26 eV, 2.31 eV, 1.40 eV, and 1.59 eV, respectively. As a result, the Fe_9_O_12_ cluster was selected to be a representative model to study the binding interactions of MCB and ERY.

Previous density functional theory (DFT) research has identified the adsorption mechanism that the magnetically modified bentonite can significantly improve the capability for adsorbing methylene blue through enhancing van der Waals forces and hydrogen bonding [33]. In this study, we utilized DFT simulations, with the model of CB-ERY (ERY adsorption on bare CB) and CB-Fe_3_O_4_-ERY (ERY adsorption on MCB) to investigate the molecular-level binding behaviors between CB, Fe_3_O_4_, and ERY, trying to unveil the role of magnetization process on bentonite.

**Figure 10:**
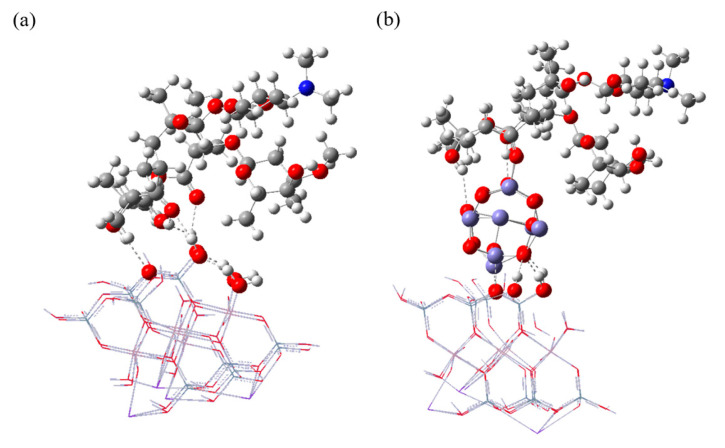
Optimized structures of (**a**) CB-ERY and (**b**) CB-Fe_9_O_12_-ERY. The hydrogen bonds are depicted using dashed lines.

The optimized structures and binding configurations of CB-ERY and CB- Fe_9_O_12_-ERY are shown in Figure 10. It can be seen that the binding behavior between CB and ERY is mainly dominated by the formation of hydrogen bonds. From a comparative perspective, the optimized structure of CB-Fe_9_O_12_-ERY demonstrated that in addition to the hydrogen bond network between CB, ERY, and Fe_9_O_12_ (the shortest H-O bond length associated with hydroxyl group of ERY is 1.697 A), the carbonyl group of ERY forms a covalent bond with Fe_9_O_12_ (the shortest Fe-O bond length associated with carbonyl group of ERY is 1.924 A). Such covalent bonding behavior had been experimentally confirmed by FTIR analysis in the system of poly(acrylic acid)-Fe_3_O_4_ [34].

#### 2.6.2. Frontier Orbital and Binding Energy Analysis

**Figure 11:**
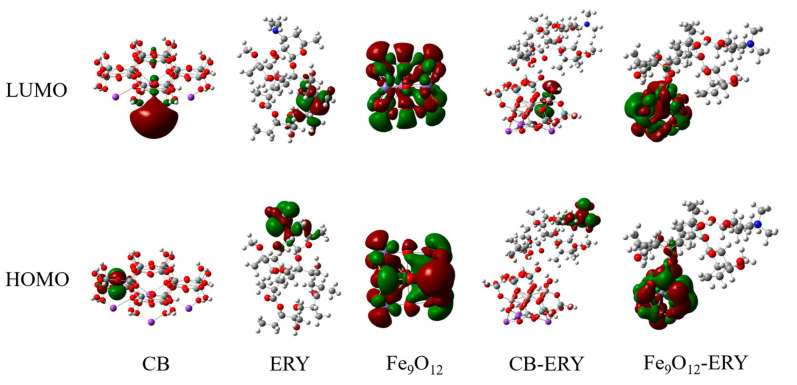
Electron distributions of the HOMO/LUMO orbitals of selected molecules.

The electron density distribution of frontier orbitals (HOMO and LUMO) can be important information to explain molecular binding interactions. The strength of binding interaction between adsorbed compounds and substrates often involves HOMO/LUMO electronic density overlap. Figure 11 shows the DFT calculated HOMO/LUMO electron distribution of CB, ERY, Fe_9_O_12_, CB-ERY, and Fe_9_O_12_-ERY. For isolated ERY, the electrons of the HOMO are primarily localized near the desosamine group, while the electrons of the LUMO are concentrated in the cladinose group. After ERY binds to CB (CB-ERY), the HOMO and LUMO are located on the desosamine group of ERY and silicate of CB, respectively. By contrast, upon ERY covalent adsorption on the Fe_9_O_12_ cluster (Fe_9_O_12_-ERY), the density of HOMO and LUMO delocalized on Fe_9_O_12_, and the HOMO electrons even extended from Fe_9_O_12_ to the covalent bond formation with the carbonyl group of ERY. This result illustrates how the Fe_3_O_4_ nanoparticle promotes adsorption behavior on magnetically modified bentonite. The delocalized LUMO electron on the Fe_9_O_12_ moiety of Fe_9_O_12_-ERY implies that Fe_9_O_12_ can accept additional ERY molecules.

The DFT-calculated HOMO/LUMO energies (columns 2 and 3 of Table 7) and the HOMO-LUMO gap ($△E_{H-L}$, column 4 of Table 7) provide insight into the electronic structure, as orbitals coupling causes energy levels splitting. The HOMO energy of CB-ERY is −5.49 eV, indicating that orbital coupling results in a 0.21 eV energy level shift when ERY is attached to CB. By comparison, ERY binding to CB-Fe_9_O_12_ results in a 0.35 eV energy level shift in HOMO orbital coupling, since the HOMO energy of CB-Fe_9_O_12_-ERY is −4.32 eV and that of CB-Fe_9_O_12_ is −4.67 eV. The calculated binding energy ($E_{b}^{ERY}$, column 5 of Table 7) of CB-Fe_9_O_12_-ERY is 2.38 eV, which is greater than that of $E_{b}^{ERY}$ of CB-ERY, indicates that the effect of the magnetic Fe_3_O_4_ nanoparticle on CB can enhance electronic coupling with ERY, resulting in greater binding energy and also ERY removal efficiency improvement.

## 3. Materials and Methods

### 3.1. Chemicals and Materials

The bentonite utilized in this study was obtained from Peilin Enterprise, located in Changhua County, Taiwan. The primary constituents of the bentonite are silica and aluminum, with particle sizes under 100 mesh (<0.149 mm). Methanol (≥99.9% purity) was sourced from Honeywell Specialty Chemicals (Morristown, NJ, USA). Sodium hydroxide and hydrochloric acid, used for pH adjustment of the aqueous solution, were procured from Fisher Chemical (Waltham, MA, USA) and Scharlau (Barcelona, Spain), respectively. Iron(III) chloride hexahydrate (≥99% purity) and iron(II) chloride tetrahydrate (≥99% purity) were supplied by Acros Organics (Geel, Belgium). Erythromycin was obtained from AK Scientific (Union City, CA, USA). All chemicals were of analytical grade, requiring no further purification prior to use in this study.

### 3.2. Preparation of Adsorbent

The thermal activation of bentonite enhances the stability and ion exchange capacity of the material, with temperature control playing a crucial role in optimizing its adsorption performance [35]. In this study, we also consider activation time as an essential parameter influencing adsorption, particularly from the perspectives of energy efficiency and carbon footprint reduction. To this end, the effects of both thermal activation temperature (400–600 °C) and activation duration (2–8 h) on the adsorption of ERY by bentonite were systematically investigated, based on optimal conditions for bentonite heat treatment. The experimental results are presented in Table 8. At a constant activation time of 4 h, ERY removal efficiency increased with rising activation temperature, aligning with previous reports. However, when the activation time was reduced to 2 h, the removal efficiency initially increased with temperature but began to decline once the temperature exceeded 500 °C. In general, prolonged activation times under a fixed temperature led to a reduction in adsorption efficiency. This can be attributed to structural degradation, as prolonged heating or higher temperatures induce dehydration and dehydroxylation processes, which disrupt the crystal structure of bentonite and diminish its adsorption capacity [18]. Based on these findings, an optimal activation condition of 500 °C for 2 h was identified.

Following the heat treatment, the calcined bentonite (CB) was cooled and stored in a sealed container at room temperature. Magnetic calcined bentonite (MCB) was synthesized via chemical co-precipitation, following the protocol outlined by Hong and Wang [36]. Specifically, 2 g of calcined bentonite, 2.7 g of ferric chloride hexahydrate (FeCl_3_·6H_2_O), and 1.2 g of ferrous chloride tetrahydrate (FeCl_2_·4H_2_O) were dispersed in 100 mL of deionized water. The pH of the suspension was adjusted to 11 using 1 M NaOH, and the mixture was heated to 85 °C while stirring at 100 rpm for 1 h in a constant temperature shaking bath. The resulting suspension was then filtered and washed with deionized water until a neutral pH was achieved. The product was dried at 50 °C overnight to yield the MCB, which was subsequently stored in a sealed container at room temperature. Although MCB was synthesized at pH 11, its magnetic properties were further analyzed under different pH conditions (pH 3, 7, and 11) to evaluate the impact of environmental pH on its magnetization behavior. This additional investigation aimed to provide insights into how variations in pH may influence the stability and applicability of MCB in different adsorption environments.

### 3.3. Material Characterization

The material characterization techniques used in this study include scanning electron microscopy (SEM, Hitachi High-Tech Corporation, Tokyo, Japan) for morphological analysis, X-ray diffraction (XRD, D8 DISCOVER SSS, Bruker Corporation, Billerica, MA, USA) for phase identification, superconducting quantum interference device vibrating sample magnetometer (SQUID-VSM, MPMS7, Quantum Design Inc., San Diego, CA, USA) for magnetic property measurements, nitrogen adsorption–desorption isotherms (BET, ASAP 2020 model, Micromeritics Inc., Norcross, GA, USA) for specific surface area analysis, and Fourier transform infrared spectroscopy (FTIR, Spectrum 100 model, Perkin Elmer Inc., Hopkinton, MA, USA) for functional group identification.

### 3.4. Batch Adsorption Study

The batch adsorption study was conducted to evaluate the effects of various operating parameters on the adsorption efficiency of ERY. The parameters investigated included solution pH (3, 5, 7, 9, 11), adsorbent dose (0.5, 1.0, 1.5, 2.0, 2.5 mg/L), adsorption temperature (25, 30, 35, 40 °C), initial ERY concentration (10, 30, 50, 70, 90 ppm), and contact time (0–24 h). In the adsorption experiments, a predetermined amount of adsorbent was added to an ERY solution of known concentration. The mixture was then placed in a temperature-controlled water bath and agitated at 100 rpm. The ERY concentration before and after adsorption was determined by measuring the absorbance at 288 nm using a UV spectrophotometer (Thermo Scientific, Ettligen, Germany). The adsorption amount (*q*) of the adsorbent and the ERY removal efficiency (R%) were calculated based on the change in absorbance, utilizing the calibration curve. The calculations followed the methodologies outlined in previous research [37].

### 3.5. Experimental Design and Statistical Analysis

To optimize the adsorption conditions and identify the key factors influencing ERY removal, this study employed the response surface methodology (RSM). RSM was chosen for its efficiency in simultaneously optimizing multiple variables and reducing the number of experimental trials required to achieve the optimal response. A central composite design (CCD) was utilized to investigate the effects of three independent variables, adsorbent dosage (A), temperature (B), and contact time (C), on the ERY removal efficiency (Y). The experimental design included five levels for each variable (−α, −1, 0, 1, +α), resulting in a total of 20 experimental runs. The relationship between the independent variables and the response variable was modeled using a second-order polynomial equation. Analysis of variance (ANOVA) was performed to assess the statistical significance of the model terms and to validate the adequacy of the fitted model. The design of the experimental matrix, optimization of the variables, regression analysis of the mathematical model, and generation of three-dimensional response surface plots were conducted using Design-Expert software (version 13). This approach enabled the identification of the optimal conditions for maximum ERY adsorption, providing a robust and statistically validated model for practical application.

### 3.6. Theoretical Investigation

In this evaluation, density functional theory (DFT) calculations were performed to investigate the binding mechanism of the ERY binding onto the bentonite and magnetically modified bentonite. The frontier orbital analysis, HOMO/LUMO positions, HOMO-LUMO gap ($△E_{H-L}$), and binding energies of ERY on various adsorbents ($E_{b}^{ERY}$) were determined by DFT calculation using Gaussian 16 [38]. The geometry optimization and frequency calculation were performed at the B3LYP/6-31G(d) level. The equilibrium geometry is ensured to be in a minimal energy state by checking the image frequency number of the corresponding structure.

The bentonite structure is generally composed of two silica sheets sandwiching one inorganic cation interlayer [39]. In this work, a K-bentonite crystal structure [40] is selected to represent calcined bentonite (CB) adsorbent. A CB cluster model is then created by extracting a finite fragment from the periodic crystalline structure of K-bentonite. To simulate the magnetization effects of CB on ERY removal, the bentonite surface is attached with the Fe_3_O_4_ nanoparticle, and a similar approach has been applied to study methylene blue adsorption behavior by magnetic bentonite [33]. Various sizes of Fe_3_O_4_ nanoparticles (Fe_3_O_4_, Fe_6_O_8_, Fe_9_O_12_ and Fe_12_O_16_) are considered to study the quantum effects of the Fe_3_O_4_ nanoparticle, since it was well known that the band gap of nanoparticle decreases while particle size increases [41].

The binding energy ($E_{b}^{ERY}$) between the ERY and CB was evaluated via the following expression:

(2) $$E_{b}^{ERY}=-\left(E_{CB-ERY}-E_{ERY}-E_{CB}\right)\;\mathrm{or}\;-\left(E_{CB-{Fe}_{3}O_{4}-ERY}-E_{ERY}-E_{CB-{Fe}_{3}O_{4}}\right)$$

where $E_{CB-ERY}$, $E_{ERY}$, and $E_{CB}$ are the electronic energies correlated with the CB−ERY, ERY, and CB complexes, respectively. $E_{CB-{Fe}_{3}O_{4}}$ represents the electronic energy of the bentonite surface modified by the Fe_3_O_4_ particle. The binding energy shows positive values, highlighting the spontaneous processes associated with ERY binding to CB. The HOMO (highest occupied molecular orbital) energy, LUMO ((lowest unoccupied molecular orbital) energy, HOMO-LUMO gap, $△E_{H-L}$, the difference between the HOMO and LUMO energies), and electron distribution of HOMO/LUMO can help to clarify the nature of the molecular interaction and quantum-size effects of Fe_3_O_4_ nanoparticles.

## 4. Conclusions

In this study, we successfully optimized the adsorption of ERY using a bentonite-based magnetic adsorbent through a simple heat treatment process followed by magnetic modification. The prepared adsorbent demonstrated high efficiency in removing ERY from water, with adsorption kinetics following the pseudo-first-order model and adsorption isotherms aligning with the Dubinin–Radushkevich (D–R) model. The calculated adsorption energy indicates that the adsorption process is primarily physical. Response surface methodology (RSM) was employed to optimize the adsorption process, considering key factors such as adsorbent dosage, temperature, and contact time. The optimal conditions—41.9 mg of adsorbent, 29.1 °C, and 9.6 h of contact time—yielded a predicted ERY removal rate of 93.7%, with an experimental value of 96.2%, confirming the accuracy of the model and the effectiveness of RSM for process optimization. The frontier orbital and binding energy analysis obtained from DFT calculations verify the covalent adsorption of ERY on magnetically modified bentonite. This work highlights the potential of heat-treated emissions. For future research, further investigations into the long-term stability and regeneration performance of the adsorbent are recommended to ensure practical applicability. Additionally, exploring composite materials with enhanced selectivity and adsorption capacity can further improve performance. Advanced spectroscopic techniques, such as XPS and in situ FTIR, could be employed to gain deeper insights into adsorption mechanisms. Lastly, pilot-scale studies should be conducted to evaluate the feasibility of large-scale applications in wastewater treatment systems.

## Figures and Tables

**Table 1:** Surface characteristics of the adsorbents.

Samples	Surface Area (m^2^/g)	Pore Volume (cm^3^/g)	Pore Size (nm)
CB	41.4	0.003	11.9
MCB	61.6	0.008	7.6

**Table 2:** Isotherm adsorption model parameters for ERY onto MCB.

Parameters	Equations	25 °C	30 °C	35 °C	40 °C
**Langmuir**	$q_{e}=\frac{K_{L}q_{m}C_{e}}{1+K_{L}C_{e}}$				
*q_m_* (mg/g)		9.93	11.53	46.73	1.31
*K_L_* (L/g)		0.708	1.051	0.077	0.050
*R_L_*		0.124	0.087	0.564	0.665
*R* ^2^		0.994	0.981	0.914	0.622
**Freundlich**	$q_{e}=K_{F}C_{e}^{1/n}$				
*K_F_* (mg/g)(L/mg)^1/*n*^		4.51	4.98	3.55	0.001
*n*		9.337	3.334	1.270	0.287
*R* ^2^		0.831	0.712	0.974	0.896
**D–R model**	$lnq_{e}=lnq_{m}-\beta \varepsilon^{2}$ $\varepsilon =RTln\left(1+\frac{1}{C_{e}}\right)$ $E=1/\sqrt{2\beta }$				
*q_m_* (mg/g)		9.24	11.66	17.49	29.40
E (kJ/mol)		1.061	0.945	0.798	0.105
*R* ^2^		0.972	0.971	0.936	0.960

**Table 3:** Kinetic parameters for ERY adsorption onto MCB.

Model	Kinetic Parameters	ERY
	*q_exp_* (mg/g)	13.36
Pseudo-first-order	*k*_1_ (1/min)	0.013
	*q_e_* (mg/g)	13.4
	*R* ^2^	0.989
Pseudo-second-order	*k*_2_ (g/mg·min)	1.3 × 10^−3^
	*q_e_* (mg/g)	14.7
	*R* ^2^	0.959

**Table 4:** Matrix for the CCD with the respective values.

Run	Independent Variables			Responses
	A: MBC Dosage (mg)	B: Temperature (°C)	C: Time (h)	ERY Removal Efficiency (%)
1	30	25	6	88.1
2	18.1	40.9	9.6	45.2
3	50	35	6	88.1
4	18.1	40.9	2.4	52.4
5	30	35	0	2.4
6	41.9	29.1	9.6	90.5
7	30	35	6	73.8
8	41.9	29.1	2.4	85.7
9	18.1	29.1	2.4	50.0
10	30	35	6	73.8
11	30	35	6	73.8
12	30	35	6	73.8
13	10	35	6	59.5
14	41.9	40.9	2.4	16.7
15	30	35	12	21.4
16	41.9	40.9	9.6	26.2
17	30	45	6	57.1
18	18.1	29.1	9.6	85.7
19	30	35	6	73.8
20	30	35	6	73.8

**Table 5:** ANOVA analysis for the quadratic model.

Source	DF	SS	*F* Value	*p* Value
Model	9	11,969.90	10.99	0.0004
A	1	83.58	0.6906	0.4253
B	1	3657.29	30.22	0.0003
C	1	410.76	3.39	0.0952
AB	1	1133.83	9.37	0.0120
AC	1	25.49	0.2106	0.6561
BC	1	181.45	1.50	0.2488
A^2^	1	16.33	0.1350	0.7210
B^2^	1	5.97	0.0494	0.8286
C^2^	1	6248.17	51.63	<0.0001
Residual	10	1210.21		
Lack of fit	5	1210.21		
$R^{2}$ = 0.9082				
Adj $R^{2}$ = 0.8255				

**Table 6:** HOMO/LUMO level, HOMO–LUMO gap ($△E_{H-L}$), and binding energy ($△E_{b}^{\mathrm{HRY}}$) of ERY adsorption on the (Fe_3_O_4_)n nanoparticle cluster.

Compound	HOMO (eV)	LUMO (eV)	$△E_{H-L}(eV)$	$E_{b}^{ERY}(eV)$
ERY	−5.28	−0.71	4.57	-
Fe_3_O_4_	−5.80	−3.54	2.26	-
Fe_3_O_4_-ERY	−5.24	−2.51	2.73	4.03
Fe_6_O_8_	−6.41	−4.10	2.31	-
Fe_6_O_8_-ERY	−5.32	−3.33	1.98	2.22
Fe_9_O_12_	−5.46	−4.06	1.40	-
Fe_9_O_12_-ERY	−4.79	−3.23	1.55	3.00
Fe_12_O_16_	−5.53	−3.94	1.59	-
Fe_12_O_16_-ERY	−4.50	−2.79	1.71	3.62

**Table 7:** HOMO/LUMO level, HOMO–LUMO gap ($△E_{H-L}$), and binding energy ($△E_{b}^{\mathrm{HRY}}$) of ERY adsorption on the CB and CB-Fe_9_O_12_.

Compound	HOMO (eV)	LUMO (eV)	$△E_{H-L}(eV)$	$E_{b}^{ERY}(eV)$
ERY	−5.28	−0.71	4.57	-
CB	−6.32	−1.57	4.76	-
CB-ERY	−5.49	−1.50	3.99	1.60
CB-Fe_9_O_12_	−4.67	−2.92	1.75	-
CB-Fe_9_O_12_-ERY	−4.32	−2.75	1.56	2.38

**Table 8:** The ERY removal efficiency (%) by bentonite was modified by different thermal procedures.

	T (°C)		
Time (h)	400	500	600
2	27.3%	44.7%	20.7%
4	~0	10.0%	40.7%
8	~0	~0	12.7%

## Data Availability

The data presented in this study are available in this article.
